# 949. Reducing Ventilator-associated Pneumonia Through Multidisciplinary Quality Improvement Initiatives

**DOI:** 10.1093/jbcr/irag033.516

**Published:** 2026-04-06

**Authors:** Brian B Draper, Liz J Day

**Affiliations:** Mercy Hospital, Springfield, MO; Mercy Hospital, Springfield, MO

## Abstract

**Introduction:**

According to the American Burn Association, mechanically ventilated burn patients are at particularly high risk for developing ventilator-associated-pneumonia (VAP). In 2022, a high incidence of VAP was observed at our hospital, placing the trauma program in the 10th decile according to Trauma Quality Improvement Program data. This prompted a comprehensive evaluation and implementation of targeted interventions to improve outcomes in ventilated patients.

**Methods:**

A multifaceted strategy was employed to enhance VAP prevention. Standardized documentation and validation by a designated trauma program member ensured accurate tracking. Providers were guided to consistently utilize a respiratory care protocol for intubated patients. Daily multidisciplinary rounds emphasized timely decisions regarding extubation or tracheostomy. Nursing staff introduced structured care cards to monitor oral hygiene, prophylaxis, sedation breaks, and equipment assessments. Audits were conducted using a visual accountability board system to reinforce compliance and enable immediate corrective actions. A Ventilator-Associated Event Committee was established, consisting of representatives from nursing, infection prevention, respiratory therapy, trauma, and critical care medicine, and quality improvement. Monthly meetings facilitated data analysis, evaluation of interventions, and ongoing staff education.

**Results:**

This collaborative approach improved adherence to evidence-based practices and supported sustained quality improvements. Initially ranted in the 10th decile for VAP incidents there was a marked reduction in the observed percentage of patients with VAP with each reporting period—from an initial rate of 3.3% to 0.4% in most recent reporting period.

**Conclusions:**

Collaborative interdisciplinary efforts, supported by structured and data-informed strategies, led to significant reductions in VAP rates and elevated the overall quality of care for mechanically ventilated patients.

**Applicability of Research to Practice:**

This model demonstrates the effectiveness of systematic, team-based approaches in reducing ventilator-associated pneumonia complications and may be adapted to other critical care settings.

**Funding for the study:**

N/A.

